# Purinergic Receptor (P2X7R): A Promising Anti-Parkinson’s Drug Target

**DOI:** 10.34172/apb.43206

**Published:** 2024-12-18

**Authors:** Saivarshini Magham, M. Lalith Kumar, Praveen Thaggikuppe Krishnamurthy, Neenu Shaji, Aishwarya Reddy Ramakkamma

**Affiliations:** ^1^Department of Pharmacology, JSS College of Pharmacy, JSS Academy of Higher Education & Research, Ooty-643001, The Nilgiris, Tamil Nadu, India.; ^2^Department of Pharmaceutics, JSS College of Pharmacy, JSS Academy of Higher Education & Research, Ooty-643001, The Nilgiris, Tamil Nadu, India.

**Keywords:** PD, P2X7R, ATP, Microglia, CNS, Allosteric antagonists

## Abstract

**Purpose:**

Parkinson’s disease (PD) is the fourth most common neurodegenerative disorder, characterized by degeneration of basal ganglia and a decrease in dopamine levels in the brain. Purinergic 2X7 receptors (P2X7Rs) serve as inflammation gatekeepers. They are found in both central and peripheral nervous systems (CNS & PNS), and are activated in glial cells during inflammation. Purinergic 2X receptors (P2XRs) have been extensively studied in recent decades, particularly P2X7R, because of their important role in neuroinflammation caused by selective overexpression in glial cells. As P2X7R and its selective antagonists may provide neuroprotection by preventing the release of inflammatory mediators such as IL-1, they have become a research focus in PD. The review covers structure, signalling, molecular mechanisms, neuroprotective role, and current developments of P2X7R antagonists in PD.

**Methods:**

A systematic analysis and review of the potential prospects of P2X7R antagonists in the treatment of PD were conducted by analyzing existing research data and reports published between 1996 and present.

**Results:**

There is a substantial body of evidence linking P2X7R to pathology of PD. As a result, P2X7R antagonists may have therapeutic potential in treatment of PD.

**Conclusion:**

P2X7R has been demonstrated as an efficacious target in PD. Recent advances in rational drug design have paved the way for development of therapeutically valuable P2X7R antagonists such as adamantyl cyanoguanides, small molecular weight compounds, and PET ligands for the treatment of PD. However, the exact molecular mechanism and therapeutic potential of P2X7R antagonists in treatment of PD are yet to be fully explored.

## Introduction

 Around ten million individuals worldwide suffer from Parkinson’s disease (PD). PD affects roughly 30 million persons in India, or 2394 people per lakh.^1^ The illness is distinguished by an aberrant build-up of synuclein (asyn) in the form of Lewy bodies, as well as a loss of dopaminergic neurons in the substantia nigra.^2^ Symptoms of PD include bradykinesia, tremor, stiffness, and postural instability. Based on the incidence of the disease in various age groups, PD is divided into three types: early-onset PD (EOPD), young-onset PD (YOPD), and juvenile. EOPD accounts for 3%–5% of all PD worldwide. Males are thought to be more susceptible to PD than females.^3^ Levodopa and other dopaminergic medicines, such as monoamine oxidase B (MAO-B) inhibitors, catechol-o-methyltransferase (COMT) inhibitors, and others, are being used to treat PD.^2^ They just relieve symptoms; they do not cure or prevent the condition. They are often administered in the early stages of the illness. Patients become even more crippled and suffer as a result of dopa-treated motor symptoms such as stooped posture, gait, and balance problems, as well as non-motor symptoms such as disturbed sleep, mood swings, cognitive impairment, pain, and drug-related side effects such as psychosis, dyskinesia. Deep brain stimulations, restoration treatments including gene therapy, glial cell-derived neurotropic factor (GNDF), and stem cell therapy, are costly and not available to all patients.

 Purinergic receptors are widely recognised for their therapeutic involvement in several disorders, including PD, Alzheimer’s disease, Multiple Sclerosis, Huntington’s disease, cancer, rheumatoid arthritis, and ischemia.^4-7^ Researchers investigated the function of extracellular adenosine triphosphate (ATP) in several cell types and origins of purinergic neurotransmission in 1976. The two primary classes were purinergic receptor 1 (P1) and purinergic receptor 2 (P2). Burnstock and Kennedy categorised P2 as P2X and P2Y based on its pharmacological action in 1985.^8^ P2X is an ionotropic ligand-gated receptor with seven subtypes (P2X1-P2X7), whereas P2Y is a G protein-coupled receptor (GPCR). P2X7 receptors (P2X7Rs) are located throughout the body but, activates when a pathological state exists. Initially, they were considered to be present solely in hematopoietic cells (macrophages, monocytes, dendritic cells, lymphocytes, erythrocytes, mast cells & eosinophils).^9^ However, they were eventually detected in osteoblasts, fibroblasts, endothelium and epithelial cells, and the central nervous system (CNS). They are present in glial cells (microglia, astrocytes) and fibroblasts in the CNS.^10^

 Microglia are immune cells that get activated as a result of pathologic circumstances such as inflammation, apoptosis, and infection. P2X7R is overexpressed in a variety of clinical diseases including PD, Alzheimer’s disease, and others.^11,12^ When a cell is inflamed or wounded, it produces a huge amount of ATP, which activates the P2X7R in microglia. Pathogen-associated molecules (PAMPs) and danger-associated molecules (DAMPs) are recognised by pattern recognition receptors in microglia (DAMPs). When ATP (a DAMP) triggers P2X7R, K + efflux occurs, which activates inflammatory mediators and induces neuronal inflammation and cell death. As a result, blocking P2X7R will be crucial in lowering inflammatory mediators and avoids neuronal cell death.^13^

 P2X7R antagonists have been proven in several preclinical models to be useful in the treatment of different neurodegenerative disorders, including PD.^14-17^ P2X7R activation has been linked with few side effects since its expression in healthy tissue is low in contrast to pathological circumstances, therefore selective targeting is required to overcome those consequences. Several P2X7R antagonists are being explored in clinical studies to treat osteoarthritis, Rheumatoid arthritis, depression, and neurological illnesses. We address the function of P2X7R antagonists as a therapeutic possibility for the treatment of PD in this review.

## P2X receptors (P2XRs)

 The first complementary deoxyribonucleic acid (cDNA) coding for P2X subunits was recovered in 1994. P2XRs can occur as homotrimers or heterotrimers, and there are seven different subtypes ranging from P2X1 to P2X7 (Table 1).^18^ They are expressed in Glial cells, neurons, epithelium, endothelium, bone, muscle, immune cells, and hemopoietic tissue.^19,20^ All P2XRs are permeable to tiny monovalent cations. The activity ranges of P2X1R and P2X3R are milliseconds and desensitise fast, but the activity ranges of P2X2R, P2X4R, and P2X6R are one second. They may also have slow or no desensitisation. P2X5R and P2X7R are unique in that they maintain a steady plateau potential through inward current during extended ATP activation. Except for the P2X5R, which permits anions (Cl-) to pass, all P2XRs allow cation inflow (Na + & Ca2 + ) and are implicated in K + outflow. P2XRs are involved in several pathological disorders including PD, Alzheimer’s disease, pain, inflammation, osteoporosis, Multiple Sclerosis, spinal cord injury, bladder dysfunction, depression, and anxiety.^21-25^ Among all the subtypes of P2XRs, P2X7R is one of the interesting targets in neurodegenerative diseases in recent times.^17^

**Table 1:** Purinergic receptor (P2XR) and its subtypes, functions, agonists, and antagonists^26^

**Family**	**Subtype**	**Receptor type**	**Endogenous agonist**	**Function**	**Potent agonists**	**Antagonists**
P2 P2X	P2X1	Ionotropic	ATP	Permeable to Na^+^, Ca^2+^, K^+^	BzATP, 2-MeSATP, ATP	NF-449, IP5I
	P2X2				ATP, 2-MeSATP	RB2, iso-PPADS
	P2X3					TNP-ATP
	P2X4				ATP	5-BDBD
	P2X5			Permeable to Cl -	ATP	BBG
	P2X6					
	P2X7			Permeable to Na^+^, Ca^2+^, K^+^	BzATP	BBG, JNJ-54175446, JNJ- 47965567, A-74003, KN-62

Adenosine tri phosphate (ATP), 2-methylthio ATP (2-MeSATP), (2’(3)-o-(4-benzoylbenzoyl) adenosine 5’-triphosphate (BzATP), Pyridoxal-phosphate-6-azophenyl-2’,4’-disulfonic acid (PPADS), Brilliant blue G (BBG), 2’3’-0-Trinitrophenyl-adenosine-5’- triphosphate (TNP-ATP), triethylammonium salt, 5-(3-Bromophenyl)-1,3-dihydro-2H-benzofuro[3,2-e]-1,4-diazepin-2-one (5-BDBD).

###  P2X7R

**Figure 1:**
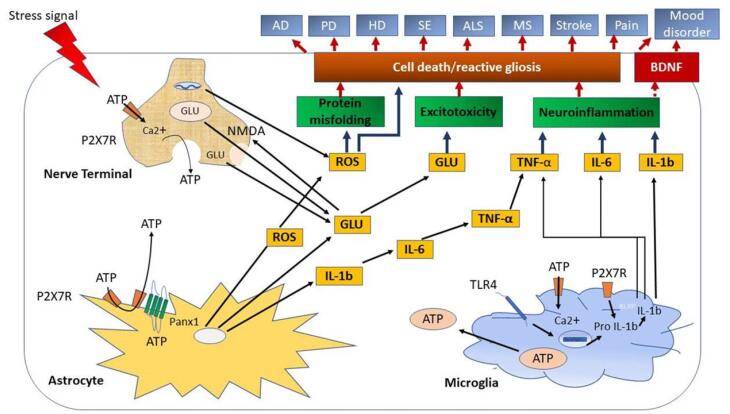
Role of P2X7R in various neurological diseases. Glutamate (GLU), N-Methyl-D-aspartic acid (NMDA), Panx1 hemichannel (Panx1), Amyotrophic lateral sclerosis (ALS), Brain-derived neurotrophic factor (BDNF), Reactive oxygen species (ROS), Intraleukin6 (IL-6), Intraleukin1-b(IL1b), Toll-like receptor 4 (TLR4), Tumour necrosis factor-alpha (TNFα)

 P2X7R expression was investigated using Northern blotting and reverse transcription-polymerase chain reaction (RT-PCR). They are located in immune system, such as spleen, as well as other organs such as the brain, spinal cord, skeletal muscles, and lungs.^20^ They are present in cortex, hippocampus, brainstem, nucleus accumbens, glial cells (astrocytes and microglia), retinal ganglia, and spinal cords of rats and humans.^27^ Because less ATP is accessible under normal settings, the P2X7R is expressed at low levels and so has restricted activity.^28^ Extracellular ATP levels rise as a result of factors such as inflammation, necrosis, and cell damage. As a result, P2X7R expression is enhanced in glial cells such as microglia and astrocyte.^29^ P2X7R is selectively expressed and increased in diseases such as PD, epilepsy, and Multiple Sclerosis (Figure 1).^30,31^ This P2X7R expression indicates that it may be useful in the treatment of neurodegenerative disorders.

###  Signaling of P2X7R

 Calcium influx is detected after P2X7R activation, which is followed by a range of cellular responses based on the cell type encountered, such as nerve terminals, astrocytes, microglia, oligodendrocytes, and so on.^32^ When P2X7Rs in nerve terminals are triggered, glutamate is released by a number of pathways that can be exocytic or non-exocytic, resulting in cell death or reactive gliosis (Figure 1). Microglia are immune cells found in the brain that works as the brain’s first line of defense, moving from a resting to an active state. When PAMPs and DAMPs are present in pathological situations such as inflammation, pain, and neuronal injury, they can sense danger. P2X7R is highly concentrated in microglia.^33^

**Figure 2:**
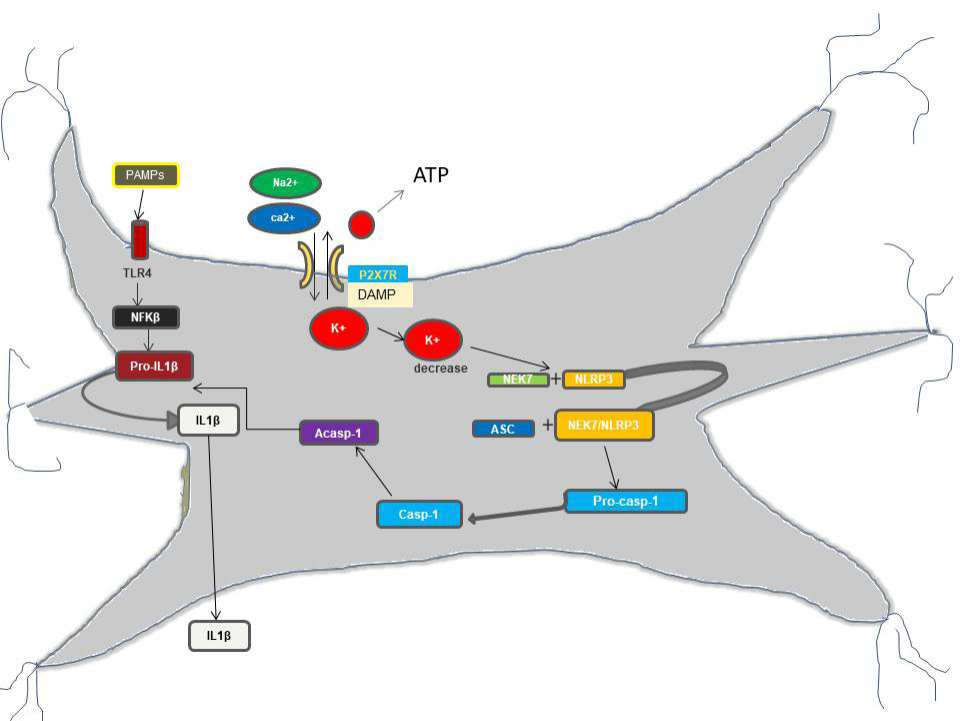
P2X7R's function in microglia. Pathogen-associated molecular protein-(PAMPs), Danger-associated molecular protein (DAMPs), Toll-like receptor 4 (TLR4), NIMA-related kinases (NEK7), nucleotide-binding leucine-rich repeat pyrin domain containing 3 (NLRP3), apoptosis-associated speck-like protein (ASC), Intraleukin1-b(IL1b), Nicotinamide adenine dinucleotide phosphate (NADPH)

 PAMPs and DAMPs on microglia detect PAMPs such as lipopolysaccharide (LPS) and ATP. Microglial cells need PAMPs and DAMPs to produce interleukin-1 (IL-1). There are two phases to the manufacturing of IL-1. TLR4 activation causes the production of pro-IL-1, which is then followed by ATP-dependent activation of P2X7R, which causes K + efflux. NIMA-related kinases (NEK7), which belong to the serine or threonine kinase family, detect a reduction in K + levels and form a complex with the inactive nucleotide-binding leucine-rich repeat pyrin domain, which has three repeats (NLRP3). By creating a multi-complex with apoptosis-associated speck-like protein, NLRP3 triggers caspase-1 conversion from pro-caspase-1 (ASC). Caspase-1-induced proteolysis converts proIL-1 to mature IL-1, resulting in inflammation (Figure 2).^34^ P2X7R induces protein misfolding and nerve damage in PD, Alzheimer’s disease, and other disorders by increasing the generation of reactive oxygen species (ROS) via nicotinamide adenine dinucleotide phosphate (NADPH) oxidase, culminating in the release of IL-6 and neuronal death.^35^

 The P2X7R in astrocytes connects pannexin hemichannels (panx1) to the inflammasome, which comprises caspase-1 and other enzymes. The inflammasome promotes the synthesis of inflammatory mediators including mature IL-1 and inflammatory cytokines like TNF-. Because NLRP3 is not present in astrocytes, it is not feasible for NLRP3 to activate IL-1 (Figure 1).^36^ Oligodendrocytes are responsible for the formation of the myelin sheath, which protects nerve axons. When P2X7Rs in oligodendrocytes are activated, they can cause white matter injury in the CNS by functioning as excitotoxic glutamate.^37^

 P2X7R has a 100 times lower affinity for agonists such as ATP than other purinergic subtypes.^38^ Despite having a low affinity for ATP, P2X7R is activated by it, although the mechanism is unknown. Under physiological settings, ATP is produced as a co-transmitter from synaptic vesicles and activates P2X7R in the autonomic nervous system (ANS). Furthermore, P2X7R activation is common in pathogenic situations. Pathological situations such as trauma, tissue injury raise the extracellular concentration of ATP, resulting in P2X7R activation.^39^ P2X7R is activated in the CNS by the release of neurotransmitters, particularly glutamate.^40^

###  Structure and molecular mechanism of P2X7R

 Only one zebrafish P2X crystal structure (ZFP2X4.1R) has been solved so far, and it is homologous to human P2X7R. Chick P2X7R (ckP2X7R) and pandaP2X7R (pdP2X7R) crystal structures indicate 85 percent identity.^41^ They established the presence of three identical ATP binding sites. Surprisingly, pdP2X7R structure results in the finding of a completely novel allosteric binding site, which is positioned in the groove produced by two neighbouring subunits.

**Figure 3:**
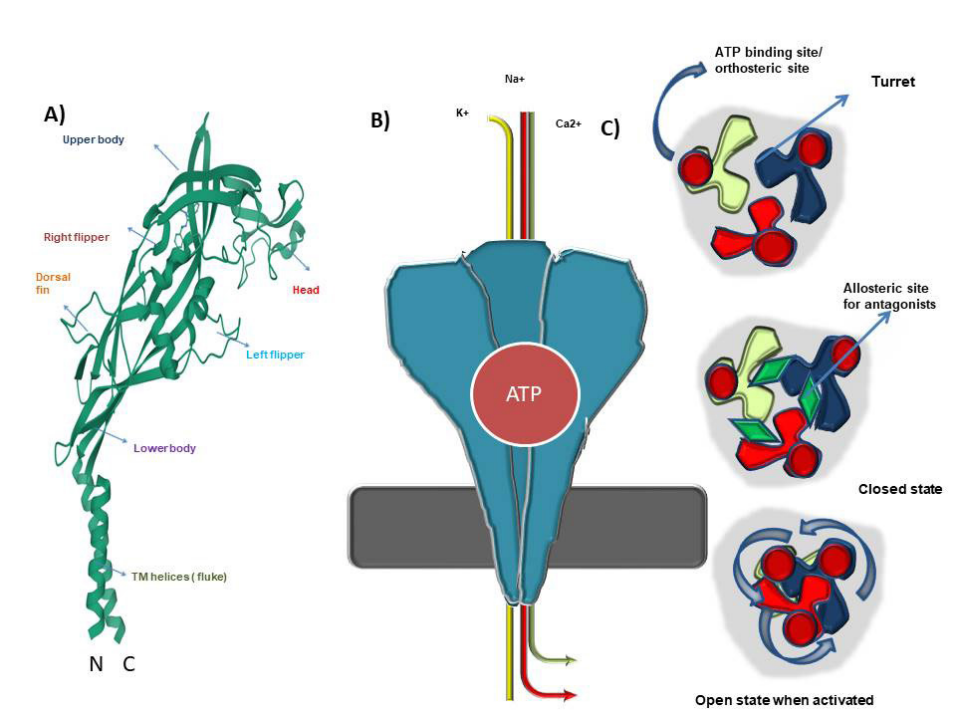
Structure and binding sites of P2X7R

 P2X7R when attached to ATP, it opens the pore channel. The bulk of mammalian P2X7R subunits is 595 amino acids long.^42^ They have two helical transmembrane (TM) domains. The intracellular N terminals, like those of other P2XR subtypes, are short, but the C terminal is 239 amino acids long. The C terminal in other P2XR variants is generally short (27-129 amino acid sequences long). The extracellular loop is made up of 269-288 longer amino acids, most of which are folded into sheets and loops.^42^ The molecular architecture of a single P2X7R mimics that of a jumping dolphin. It’s broken down into six components. 1. The realm of the mind 2. the upper body, 3. Dorsal fin, Dorsal fin, Dorsal fin, Dorsal fin, Dorsal fin 5. right flipper and 6. left flipper (Figure 3A). When a trimeric unit is produced, the extracellular loop serves as the body, while the TM domain serves as the tail (Figure 3C). P2X7R resembles a chalice structure that is joined together and participates in the creation of the channel pore (Figure 3B).^43^

 P2X7R contains three ATP binding sites. To accomplish confirmation or activation, at least two of the available three ATP sites must be occupied.^20^ When it binds to adenine-based phosphate molecules, it undergoes a conformational shift that culminates in the creation of a ‘U’ shape. When ATP connects to the pocket, it causes turret narrowing and stretching. The upper body subunit decreases towards the central axis during this process, enabling the lower body to expand and create the appropriate structural conformation for permitting ion transit away from the central axis. This conformational shift extends the transmembrane domain vertically, resulting in the channel gate opening.^43^

 When activated, P2X7R expands the channel pore, enabling tiny cations such as Ca2 + , Na2 + , and K + to flow through. One of P2X7R’s most prominent qualities is its capacity to remain engaged for a lengthy period, enabling molecules bigger than 600-800 Daltons to pass through. N-methyl-D-glutamine (NMDG + )/high molecular weight dyes such as VOPRO-1ethidium bromide can be used to explore this pore-forming feature. Two processes contribute to the receptor’s sustained activation.^44^

**Figure 4:**
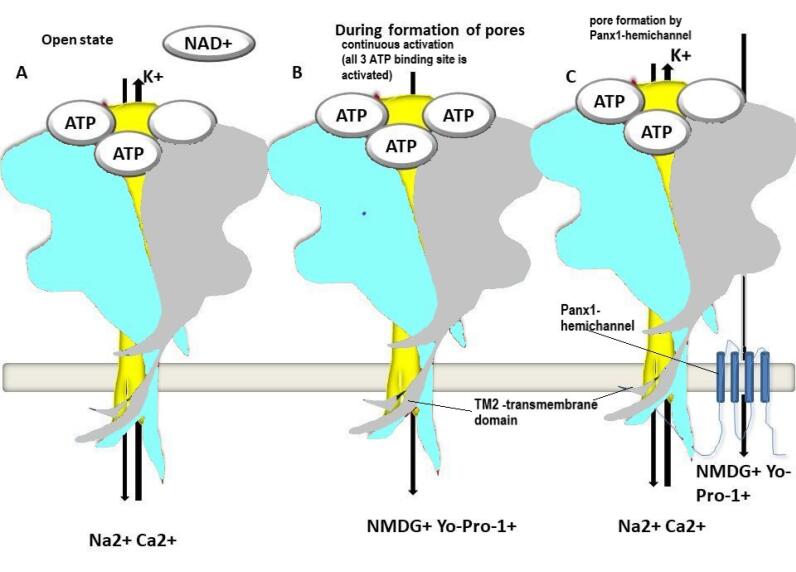
Structure of P2X7R in the open state (A) and during pore formation (B&C)

 According to the first mechanism, the receptor is engaged in ligand-gated channel dilatation during continuous activation by dyes such as NMDG, Yo-Pro-1, and others. P2X7R channel dilatation may be accomplished by conformational alteration. Pore formation is aided by the TM2 region and the carboxyterminal domain (Figure 4). Recent research, on the other hand, suggests that the open channel conformation may also accommodate negatively charged molecules with widths of 1.4 nm, such as fluorescent dyes. When one or two agonists are occupied, the transition desensitized state occurs. When the third agonist binding occurs, the transition state is dilated or sensitized (Figure 4).^41^

 The existence of a protein known as panx1 hemichannel, which generates a pore and permits big molecular weight molecules such as NMDG, Yo-Pro-1, and others to pass through, is proposed in the second mechanism (Figure 4). Pelegrin and Surprenant’s panx1 deletion experiments dramatically inhibited 2-&3-O-(4-benzoyl-benzoyl) adenosine5-triphosphate (BzATP) driven YO-PRO-1 absorption in heterogeneously expressed HEK293 cell lines employing rat P2X7R.^45^ Suadicani et al showed that panx1 mutant mice exhibited decreased YO-PRO-1 uptake in astrocytes, showing that Panx1 channels are crucial in pore formation and P2X7R activity.^46^ Panx1 opening bigger pores may cause cell death via a process involving membrane blebbing and gene alterations, resulting in P2X7R encoding and pore hypofunction. However, it may be treated with peptides that correspond to the region in the P2X7R’s C-terminus, which can block pore formation while permitting channel cation activity (Figure 4).

 P2X7R is distinguished by its extended desensitization feature. In contrast to other P2X subtypes, such as P2X1R and P2X3R, which show fast desensitization during electrophysiological studies, it shows very little or no desensitization throughout many seconds of agonist ATP activation.^47^ The P2X7R is distinguished from other purinergic subtypes by a lengthy intracellular C terminal domain of 239 amino acids. This lengthy C terminal characteristic has been crucial for the pore-forming property of the P2X7R. Surprisingly, an LPS binding site was found at this C terminus, suggesting that the receptor may be involved in the transmission of inflammatory signals.^48^

###  P2X7R allosteric binding pockets

 The allosteric site is the major targeted site for P2X7R antagonists due to its greater selectivity, reduced toxicity, and fewer side effects. At the orthosteric site, antagonists must compete with agonists to block the receptor. Nonetheless, there is no need for competition to block the receptor at the allosteric site.^49^ Despite structural variations, P2X7R antagonists such as JNJ47965567, A804598, AZ10606120, GW791343, and A740003 have been shown to bind to the allosteric site groove.^50^ Beta-sheets 4, 13, and 14 create the allosteric binding site for many antagonists. Internally, on the top of the upper body subunit, an inter-subunit cavity is produced. They keep the turret from decreasing and the channel from opening (Figure 4C).^50^ Because of the lack of the leu217 residue moiety, which is important in ribose interactions, human P2X7R has a 100-fold lower affinity for ATP than other P2XRs.^43^

 Allsopp et al employed AZ11645373, a selective P2X7R antagonist, to examine the allosteric binding site of P2X7R utilising point mutations and chimeras.^50^ According to their findings, the intersubunit is separated into three sections: the entry portion, the middle portion, and the base portion. The allosteric binding pocket of human P2X7R is composed of two positively charged lysine residues, lys110 and lys306, which are situated near the intersubunit entryway. Lys110 was discovered to have hydrogen bonds with the nitrogen group, as well as aromatic interactions with the nitrobenzene group of AZ11645373. Furthermore, nicotinic interactions with AZ11645373 were discovered for phe95 and phe103. Mutations in phe88ala, thr90val, asp92ala, phe103ala, and val312ala were also shown to reduce sensitivity in the allosteric binding site. These amino acid residues were assumed to be essential for allosteric intersubunit binding. Furthermore, the binding sites of P2X7R antagonists such as ZINC58368839, BBG, KN-62, and calmidazolium were studied. MODELER was utilised in molecular modelling using templates (PDB ids: 5U1U, 5U1V, 5U1W, 5U1X, 5U1Y), followed by Rosetta Ligand ensemble docking.

###  Binding mode of P2X7R allosteric antagonists 

**Figure 5:**
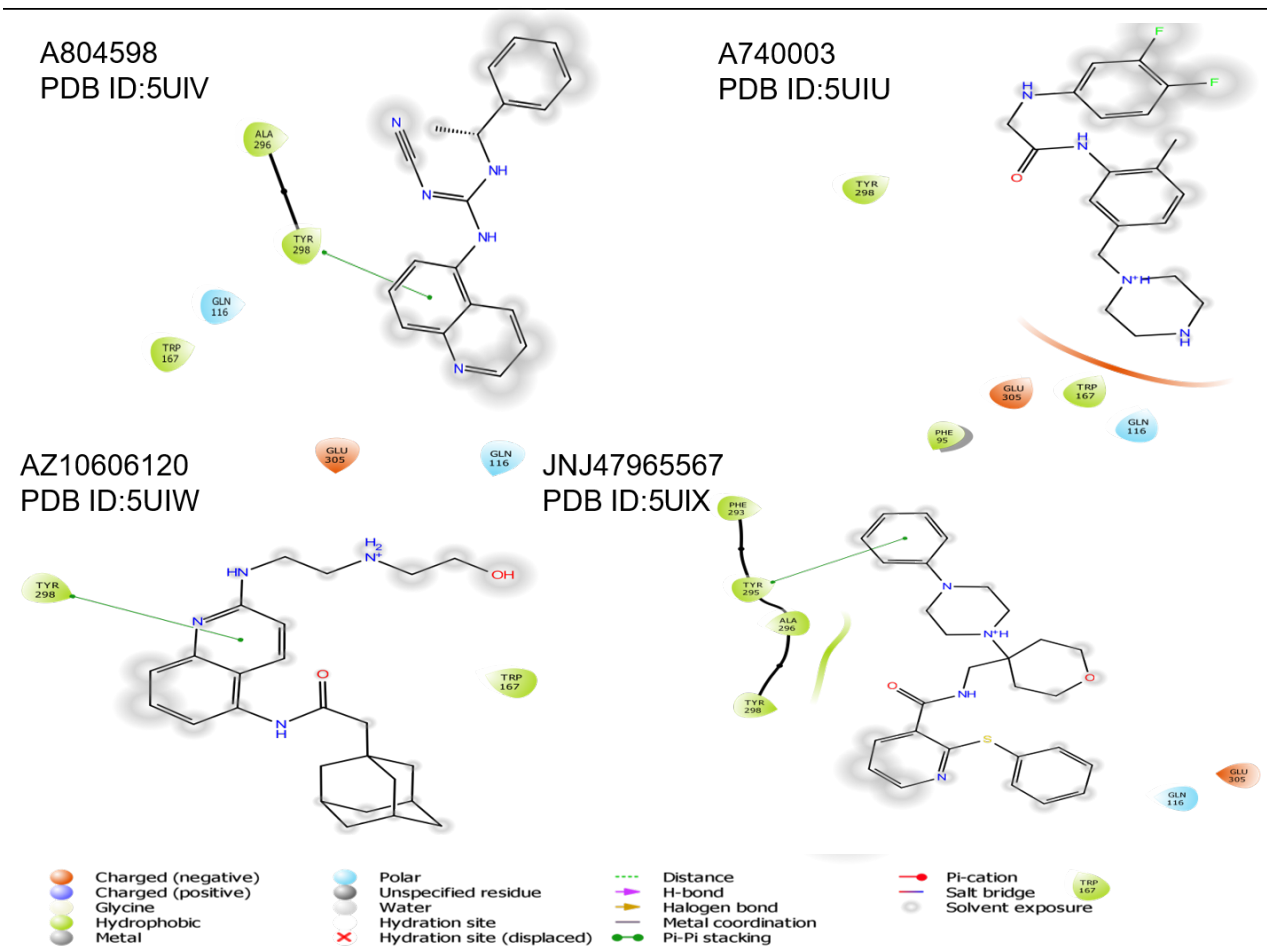
2D interaction diagrams of allosteric antagonists of P2X7R

 The amino acid interactions of P2X7R with recognised antagonists such as A804598, A74003, AZ10606120, and JNJ47965567 are given below (Figure 5). Karasawa and Kawate investigated the amino acid interactions of P2X7R with allosteric antagonists such as A804598, A74003, AZ10606120, and JNJ47965567 and discovered that all five structurally unrelated antagonists bind in the intersubunit cavity, which is surrounded by 13 residues, the majority of which are β strands (β4, β13 & β14).^51,52^ Allosteric binding is predominantly mediated by hydrophobic contacts, which are implicated in interactions with phe95, phe103, met105, phe293, and val312. The key binding residues of A740003 include phe108, phe88, tyr295, val312, met105, and phe103. The key binding residues of A804598 are phe108, phe88, tyr295, met105, phe95, and phe103. The key binding residues of AZ10606120 include phe108, val295, val312, phe293, phe103, and phe88. The key binding residues of JNJ47965567 include phe108, tyr295, val312, phe293, met105, phe103, phe95, phe88, and tyr167.

 With the exception of A740003, the amino acid sequences tyr298 and tyr295 were responsible for pi-pi stacking interactions in all of the ligands listed above.^52^ Pi-pi stacking is a non-covalent contact that arises mostly owing to the existence of parallel aromatic groups. These interactions are considered crucial since they are necessary to safeguard the drug’s structure and promote its release. As a result, having this characteristic available is useful in the creation of selective P2X7R antagonists.

## P2X7R antagonists in PD

 P2X7R antagonists are classified into two groups based on their ability to bind to the receptor. Allosteric molecules bind at the inter-subunit site, which excludes ATP binding pockets, whereas orthosteric molecules compete for the ATP site (Figure 3). In the 1990s, the first generational P2X7R antagonists were produced, including reactive blue, CAM-kinase-II inhibitor (KN-62), and oxidized ATP (OATP).^53^ Suramin and its equivalents are inert or less powerful P2X7R inhibitors when compared to other P2XR subtypes. The human P2X7R antagonists Coomassie brilliant blue G (BBG) and pyridoxal phosphate-6-azophenyl-2-4-disulfonic acids (PPADS) have pIC50s of 7.3. With the exception of BBG, all of these compounds have limitations such as poor draggability and low specificity.^54^

 BBG has become one of the most extensively utilized compounds, and it has been found in humans, rats, and guinea pigs to be efficacious.^50^ BBG inhibited inflammasome activation and reduced disruption of the Blood-Spinal cord barrier induced by spinal cord injury in rats.^55^ It also has the capacity to traverse the blood-brain barrier (BBB), but it was nonspecific because it also inhibits other P2XR subtypes such as P2X4R and P2X1R. Other P2X7R antagonists include functionally comparable compounds that block synthetic enzymes, such as alkaloids and phospholipase D inhibitors (CAY10593).

 Tetrazoles, such as A438079, was launched in 2006 as second-generation P2X7R antagonists with enhanced selectivity, followed by cyanoguanides, such as A74003.^56^ Both were expected to bind orthosterically, however, the recently discovered molecule A74003 may also bind allosterically. Following that, GSK314181A, AZ11645373, AZD9056, and CE-224, CE-535 were revealed to have high brain bioavailability and to be beneficial in numerous preclinical models of inflammation, stroke, brain damage, inflammatory bowel disease, and cancer.^57^

 The bulk of PD models involves neurotoxicity produced by toxins such as 6-OH-Dopamine (6-OHDA), 1-methyl-4-phenyl-1, 2, 3, 6-tetrahydropyridine (MPTP), LPS, and rotenone, as well as a few via deletion of genes such as parkin and asyn in mice.^58,59^ Many of the toxins stated above will cause PD by activating microglia, which leads to the production of inflammatory cytokines like TNF and IL-1, which are the fundamental causes of PD.

 P2X7R levels in microglia were raised when LPS was injected into the substantia nigra; however, pretreatment with BBG reduced microglia activation and dopaminergic neuron death.^60^ BBG decreased activated microglia in LPS-induced mice via the intranigral pathway.^61^ In a rat model of PD induced by 6-OHDA, P2X7R antagonist A438079 reduced striatal dopamine loss but had very little effect on preventing dopaminergic cell death.^16^ P2X7R antagonists were utilized in conjunction with radio-labeled markers such as [11c]JNJ-717 and [18F]DPA-714 to monitor the number of microglia after 6-OHDA treatment, and they displayed maximal binding to P2X7R.^31^ Also, [11C]JNJ54173717 has been employed in labelling and measuring the P2X7Rs in the brain.

 The above pieces of evidence show that BBG therapy has neuroprotective benefits such as reduced cognitive impairments, oxidative stress, restored brain dopamine levels, and changes in mitochondrial membrane permeability. BBG was also observed to lower oxidative stress and mitochondrial complexes, as well as alterations in mitochondrial membrane potential. In vivo studies using neuroblastoma SH-SY5Y cells revealed that P2X7Rs are triggered by the PNS directly to the release of ATP, resulting in PD.^62^ P2X7R antagonists are directly related to the kind of neurotoxic provided; for example, in a study using MPTP or the rotenone model, the deletion of the P2X7R gene failed to impress since it did not influence survival rate as predicted.^63^ Novel small compounds, such as JNJ-47965567 and polycyclic carboranes have been identified as potential antagonists and CNS active (Table 2).^64^ Compounds such as CE-224,535 and AZD9056 (Table 2) demonstrated good safety and tolerability in phase 2 studies but were ineffective in Rheumatoid arthritis patients^65^; nonetheless, they paved the door for the development of P2X7R antagonists in other areas such as CNS.

**Table 2:** P2X7R antagonists,their pharmacodynamic (PD), pharmacokinetic (PK) properties, and plases of clinical trials^4^

**Antagonist name**	**PD activity**	**PK properties**	**Clinical trials**
Oxidized ATP (oATP)	Irreversible	Only pharmacological tool	-
Pyridoxalphosphate-6- azophenyl-2,4-disulfonicacid (PPADS)	P2X selective	Not BBB permeable	-
BBG	P2X selective	BBB permeable	-
Suramin	Non P2 selective	Not BBB permeable, but circumventricular organs permeable, long half-life	-
KN-62	mP2X7R selective CaM kinase 2 inhibitor	Only pharmacological tool	-
Quinazolinium derivative	Sub-nanomolar range activity on P2X7R	-	-
N-methylindolepyrazolodiazepinone	Less potent	Better properties	-
Pyrimidine-2,4-diones	IC50 = 27 nM	High metabolic stability, low cytotoxicity and cardiac toxicity	-
N-Phenyl pyrazoles	IC50 = 10-20 nM	CYP3A4 inhibition	-
Pyro glutamic derivatives	IC50 = 3. 2 nM	Metabolic stability, short half-life (1.5 h)	-
Imidazolone derivatives	IC50 = 10 nM	Slow microsomal clearance	-
1,2,4-Triazine	IC50 = 1.1 nM	clog > 6	-
CE-224,535	IC50 = 4 nM	clog = 2.9	Phase-2-osteoaethritis Phase-3-rheumatoid arthritis
Adamantane-cyanoguanidine	IC50 = 69 nM	High clog P	-
Trifluoro-adamantane-o-chloro benzamide	IC50 = 33.9 nM	High metabolic stability, high bioavailability, short brain half-life (75.6 m)	-
Quinoline derivative	IC50 = 35 nM	Good Bioavailability	-
JNJ-47965567	hP2X7R IC50 = 5.3 Nm	Low brain half-life	-
JNJ-54166060	hP2X7R IC50 = 4 Nm	High microsomal stability CYP3A4 inhibition	-
JNJ-54175446	hP2X7R IC50 = 3 Nm	High metabolic stability and bioavailability, no CYP3A4 inhibition, low off-target effects	Phase 1 Major depressive disorder
JNJ-55308942	hP2X7R IC50 = 10 nM	High water solubility, high brain half-life (20 h in dogs)	Phase1 Healthy participants

## The recent development in P2X7R antagonists

 In recent years, researchers have focused on diverse classes of small molecular weight substances and the production of drug-like P2X7R ligands since the P2X7R has been identified as the most druggable target in the P2XR family. Compound libraries are being used to study and explore centrally penetrating powerful P2X7R antagonists, and recent advances in molecular screening technologies have aided in the production of important novel P2X7R antagonists. Teniposide is a therapeutically useful P2X7R antagonist that was produced using allosteric modulators and natural moiety analysis.^66^ Multi-targeted specific ligands (MTLDS) are new types of P2X7R antagonists that have several advantages in the treatment of neurodegenerative diseases.^67^ Recently, numerous cyanoguanides that are powerful P2X7R antagonists, such as adamantyl cyanoguanides, have been discovered.^68^ Polycyclic compounds with high efficacy and selectivity for P2X7R have been described. These compounds include aryl carbohydrazines, adamantyl isoquinolines, and adamantyl benzamides.^69^

**Figure 6:**
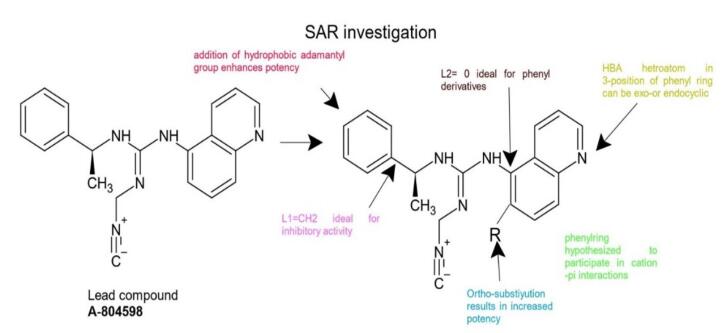
Structural activity relationship (SAR) of adamantyl cyanoguanides

 By enhancing the lipophilic ability of polar molecules, the adamantyl group boosted BBB penetration; this adamantyl group is one of the hydrophobic groups that are most powerful in the structure-activity relationship (SAR) of P2X7R antagonists (Figure 6).^69^ When compared to current phenyl analogues, the inclusion of aryl groups, which are heterocyclic compounds like AstraZeneca’s indazole 1, reduced intrinsic clearance (Clint = 47 ml/min/kg) and produced a moderately productive half-life of 1 hour in rats. Among the chemicals studied, 5-quinolinyl 27, an adamantyl methyl analogue, was shown to have the highest efficacy (18 nM). As a result, these molecules have a higher potential for generating CNS permeable P2X7R antagonists in the coming days. Recently, many molecules with more druggable properties are developing as P2X7R antagonists (Table 3).^43^

**Table 3:** Novel P2X7R antagonists^70^

**Class/Compound**	**Function**
Novel small molecules	-
(1H-pyrazol-4-yl) acetamides	Antagonist
Benzamides	Antagonist
Tetrasubstituted-imidazoles	Antagonist
2-oxo-N-(phenylmethyl)-4-imidazoline carboxamides	Antagonist
CNS active	-
JNJ - 47965567	Antagonist
Polycyclic carboranes	Antagonist
Identified by screening compound library	-
Clemastine	Positive allosteric modulator
Perazine-type antipsychotic drugs	Negative allosteric modulators
Ivermectin	Positive allosteric modulators
Natural compounds	-
Teniposide	Antagonist

## Conclusion

 P2X7R is required for the pathophysiology of CNS illnesses such as PD, stroke, neurotrauma, epilepsy, neuropathic pain, Multiple sclerosis, Alzheimer’s disease, and Huntington’s disease. As a result, P2X7R antagonists have potential therapeutic action and are believed to have few adverse effects due to the low density of P2X7Rs in healthy tissue and their expression exclusively under pathological situations. Even though some P2X7R antagonists failed to pass the blood-brain barrier, P2X7R antagonists such as BBG and P2X7Rs ligands have demonstrated their ability to traverse the blood-brain barrier. Recent breakthroughs in rational drug design have opened the way for the discovery of novel and therapeutically effective allosteric P2X7R antagonists for the treatment of PD, such as adamantly cyanoguanides, small molecular weight compounds.

## Competing Interests

 None.

## Data Availability Statement

 Data sharing is not applicable to this article as no new data were created or analyzed in this study.

## Ethical Approval

 Not applicable.
